# School-Based Mandatory Masking Policies and Absenteeism in Ottawa, Canada, in 2022

**DOI:** 10.1001/jamanetworkopen.2023.25799

**Published:** 2023-07-26

**Authors:** Nisha Thampi, Kevin L. Schwartz, Michelle Science, Kevin A. Brown

**Affiliations:** 1 Division of Infectious Diseases, Immunology and Allergy, Children’s Hospital of Eastern Ontario, Ottawa, Ontario; 2 Division of Infectious Diseases, Unity Health Toronto, Toronto, Ontario; 3 Division of Infectious Diseases, The Hospital for Sick Children, Toronto, Ontario; 4 Public Health Ontario, Toronto, Ontario

## Abstract

This cohort study assesses the association between school-based mandatory masking policies and educational disruption in Ottawa, Canada, during the COVID-19 pandemic.

## Introduction

School-based mandatory masking policies may reduce SARS-CoV-2 incidence among students and staff.^1^ On March 21, 2022, all 4 Ottawa school boards lifted mandatory masking policies in line with guidance for other indoor spaces, but the policy was reintroduced by the largest board on April 13, 2022, in response to the rising community prevalence of SARS-CoV-2. We examined whether school-based mandatory masking policies were associated with reduced educational disruptions due to SARS-CoV-2 and other respiratory viruses.

## Methods

This cohort study was exempt from review by the Children’s Hospital of Eastern Ontario research ethics board because it involved the secondary use of anonymous data. This report follows the Strengthening the Reporting of Observational Studies in Epidemiology (STROBE) reporting guideline. We estimated the association of school-based masking policies with student and staff absenteeism in public elementary (junior kindergarten to grade 8) and secondary (grades 9 to 12) schools in Ottawa from March 21 to May 19, 2022, using a difference-in-differences design. During this time, there was cocirculation of SARS-CoV-2, influenza A, and respiratory syncytial virus.^2^ We excluded 2 days for a religious holiday (May 2 and 3), adult schools, and schools that did not report absenteeism data for at least 80% of school days (32 of the 40 school-day observation period).

The exposure was the presence of a school-based mandatory masking policy, which was derived from media reports. We did not expect an immediate association given the incubation period of SARS-CoV-2 and therefore included a wash-in period, where the intervention was assumed to have no association on day 1, then increasing by 10% per school day to reach the full result after 10 school days based on the distribution of incubation periods.^3^ The outcome, student and staff absenteeism counts, was reported daily to the Ministry of Education and coded as absenteeism proportion weighted by school enrollment.^4^

The difference-in-differences logistic regression model included a separate intercept for schools with mandatory masking policy and schools with no mandatory masking policy, a cubic spline with a knot for each week of the study period, and the exposure variable.^5^ We also included a random effect for each school to capture variation in baseline absenteeism. The regression models were fitted using the lme4 package in R version 4.3.0 (R Project for Statistical Computing). We reported odds ratios (ORs) from the logistic regression model with 95% CIs. The absolute absenteeism reduction (%) and 95% CI was estimated by applying marginal standardization and parametric bootstrapping to the logistic regression model. The statistical significance threshold was *P* < .05 based on 2-sided testing.

## Results

Of 363 eligible schools, 205 (56.5%) provided adequate absenteeism data; 166 (81.0%) of the included schools were elementary schools (60 of 166 [36.1%] had mandatory masking policies), and 39 (19.0%) of the included schools were secondary schools (12 of 39 [30.8%] had mandatory masking policies). Enrollment in the included schools was estimated to be 77 640 students (Table). Reintroduction of school-based mandatory masking was associated with a reduction in absenteeism in elementary and high schools (OR, 0.96; 95% CI, 0.93-0.98; *P* = .002; absenteeism reduction, 0.4%; 95% CI, 0.1%-0.5%; 1933 school days gained) (Figure). The reduction was similar in elementary schools (OR, 0.95; 95% CI, 0.92-0.98) and secondary schools (OR, 0.96; 95% CI, 0.91-1.02; *P* = .78).

**Table.** Absenteeism in Ottawa Region Schools, March 21 to May 19, 2022

Mandatory masking policy	Schools, No.	Student and staff absenteeism, reported No. absent/total enrollment (%)			Mandatory masking policy estimates (difference-in-differences)	
		Premask policy period, March 21-April 13	Mask policy period		Odds ratio (95% CI)	Absenteeism reduction (95% CI), %
			Wash-in, April 14-April 28^a^	Result, April 29-May 19^a^		
**All schools**						
No	133	108 779/878 285 (12.4)	51 560/424 935 (12.1)	62 253/616 300 (10.1)	0.96 (0.93 to 0.98)	0.4 (0.1 to 0.5)
Yes	72	58 561/437 395 (13.4)	27 646/215 415 (12.8)	32 503/303 795 (10.7)		
**Elementary**						
No	106	77 923/637 885 (12.2)	17 962/137 775 (13)	20 427/194 670 (10.5)	0.95 (0.92 to 0.98)	0.4 (0.1 to 0.6)
Yes	60	38 298/277 830 (13.8)	14 602/112 685 (13)	19 981/168 970 (11.8)		
**Secondary**						
No	27	30 856/240 400 (12.8)	51 560/424 935 (12.1)	62 253/616 300 (10.1)	0.96 (0.91 to 1.02)	0.3 (−0.1 to 0.8)
Yes	12	20 263/159 565 (12.7)	27 646/215 415 (12.8)	32 503/303 795 (10.7)		

^a^ The mandatory masking policy was implemented on April 13, 2022. The difference-in-differences analysis specified that the estimated impacts of the mandatory masking policy washed-in incrementally over a 10 school day period from April 14 to April 28, 2022.

**Figure.**
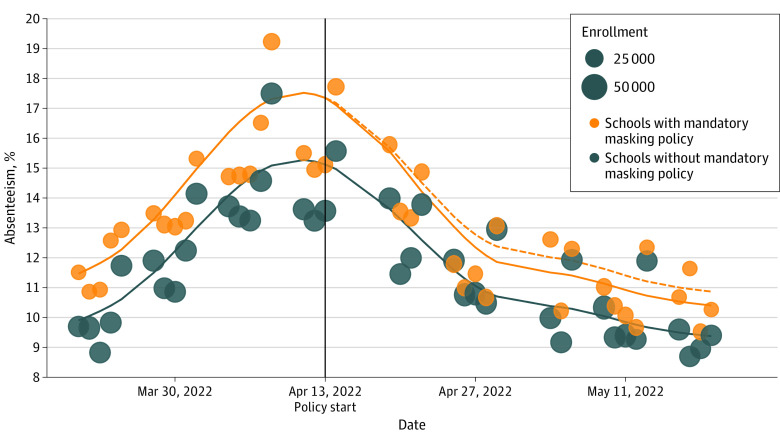
Absenteeism in Ottawa Region Schools, March 21 to May 19, 2022 Each dot represents the mean proportion of absenteeism for that day in all schools with and without a mandatory masking policy. The solid lines represent the smoothed percentage absenteeism estimates in schools with (orange) and without (blue) mandatory masking policy. The mandatory masking policy was reintroduced on April 13, 2022 (black vertical line). The difference-in-differences analysis specified that the estimated impacts of the mandatory masking policy washed-in incrementally during a 10 school day period from April 14 to April 28. The dashed orange line is the expected absenteeism in the absence of a mandatory mask policy. A reduction in absenteeism in the days following April 13 was observed in schools that implemented mandatory masking policies (blue dots: N = 72 schools; 26 440 students), compared with those that did not (orange dots: N = 133 schools; 51 200 students).

## Discussion

In this cohort study, the reintroduction of school-based mandatory masking policies during a 6-week period of sustained respiratory viral activity in Ottawa was associated with a small but statistically significant decrease in student and staff absenteeism. This study has limitations. The study design accounted for temporal confounding and secular trends but other confounding for differential changes in absenteeism between schools was possible. Only 56.5% of schools consistently reported absenteeism data during the study. The absenteeism metric was not standardized across school boards, and there was likely variability in the quality of reporting.^6^ Absenteeism reporting did not differentiate between students and staff, nor could compliance be assessed with the mandate or other preventive measures. Decisions on earlier return to school following respiratory illness may have been informed by the presence of a mandatory masking policy. During periods of high respiratory viral activity in the community, school-based mandatory masking policies may be associated with small reductions in absenteeism, possibly secondary to reduced respiratory virus transmission.

## Notes

Supplement.
Data Sharing Statement
