# Errors in the Table

**DOI:** 10.1001/jamanetworkopen.2023.34437

**Published:** 2023-09-15

In the Research Letter titled “School-Based Mandatory Masking Policies and Absenteeism in Ottawa, Canada, in 2022,”^1^ published July 26, 2023, there are errors in the data for the mask policy period. The data for yes and no under the mask policy subheading were transcribed incorrectly. This article has been corrected.^1^
