# Supplementary material for: School-Based Mandatory Masking Policies and Absenteeism in Ottawa, Canada, in 2022
Source: JAMA Netw Open. 2023 Jul 26;6(7):e2325799. doi: 10.1001/jamanetworkopen.2023.25799 (PMC10372699; doi:10.1001/jamanetworkopen.2023.25799)
Supplement: Supplement. — Data Sharing Statement [file jamanetwopen-e2325799-s001.pdf]

Data Sharing Statement

Thampi. School-Based Mandatory Masking Policies and Absenteeism in Ottawa, Canada, in 2022. *JAMA Netw Open*. Published July 26, 2023. doi:10.1001/jamanetworkopen.2023.25799

Data
Data available: Yes
Data types: Deidentified participant data
How to access data: 2022 student and staff absenteeism data were published by the Ministry of Education and are publicly available: https://data.ontario.ca/dataset/summary-of-cases-in-schools/resource/e3214f57-9c24-4297-be27-a1809f9044ba
When available: With publication

Supporting Documents
Document types: None

Additional Information
Who can access the data: Data are publicly available
Types of analyses: Data will be available for any type of analyses for any purpose
Mechanisms of data availability: Data available without investigator support
